# Antimicrobial Susceptibility Profiles of *Staphylococcus aureus* Isolates from Domestic Pigeons in Hungary in 2022

**DOI:** 10.3390/antibiotics14050525

**Published:** 2025-05-20

**Authors:** Ádám Kerek, Ábel Szabó, Ákos Jerzsele

**Affiliations:** 1 Department of Pharmacology and Toxicology, University of Veterinary Medicine, István utca 2, H-1078 Budapest, Hungary; szabo.abel@student.univet.hu (Á.S.); jerzsele.akos@univet.hu (Á.J.); 2 National Laboratory of Infectious Animal Diseases, Antimicrobial Resistance, Veterinary Public Health and Food Chain Safety, University of Veterinary Medicine, István utca 2, H-1078 Budapest, Hungary

**Keywords:** *Staphylococcus aureus*, antimicrobial resistance, minimum inhibitory concentration, MIC, pigeons, MDR

## Abstract

**Background**: Antimicrobial resistance (AMR) is a critical global health threat, affecting both human and veterinary medicine. Pigeons are increasingly recognized as potential reservoirs of antibiotic-resistant bacteria due to their widespread presence in urban and rural environments. The aim of this study was to determine the antimicrobial susceptibility profiles of *Staphylococcus aureus* isolates from pigeons in Hungary. **Methods**: A total of 73 *S. aureus* isolates were collected from pigeons across seven regions of Hungary in 2022. Minimum inhibitory concentrations (MICs) were determined using the broth microdilution method according to Clinical and Laboratory Standards Institute (CLSI) guidelines. Statistical analysis included correlation heatmaps, hierarchical clustering, network analysis, decision tree modeling, and Monte Carlo simulations. **Results**: The multidrug-resistant (MDR) prevalence rate was alarmingly high at 80.8%. Very high resistance rates were observed for doxycycline (97.3%), enrofloxacin (87.7%), and amoxicillin (84.9%). By contrast, low resistance rates were detected for vancomycin (5.5%) and imipenem (8.2%). Decision tree modeling identified tiamulin, enrofloxacin, and amoxicillin-clavulanate resistance as the most significant predictors of MDR status. Monte Carlo simulations predicted a mean MDR prevalence of 78.5%, indicating that the dominance of MDR strains is not merely a random phenomenon but part of a broader epidemiological pattern. **Conclusions**: These findings confirm that pigeons may serve as critical reservoirs of MDR *S. aureus* strains, posing a potential risk to public and animal health. Continued monitoring, the genetic characterization of resistant strains, and the development of effective control strategies are urgently needed. This study provides a foundation for future research aimed at understanding the biological, ecological, and epidemiological roles of pigeon-associated MDR strains.

## 1. Introduction

Antimicrobial resistance (AMR) poses a severe threat to the effectiveness of antibiotics, making the treatment of infectious diseases increasingly challenging and costly [1]. AMR not only complicates the management of bacterial infections but also significantly increases the risk of future pandemics [2]. Recognizing the global impact of AMR [3], the World Health Organization (WHO) has issued guidelines to promote responsible antimicrobial use [1]. Reflecting the gravity of the issue, many governments have introduced regulations and policies to limit the misuse of antibiotics [3].

Pigeons (*Columba livia domestica*) warrant special attention due to their potential role in the dissemination of AMR. While revered as sacred animals in parts of Asia [4], and symbolizing fertility in Mediterranean cultures [5], pigeons have historically served various functions, including communication during wartime [6]. Today, they are bred for sport, companionship, and meat production [4]. Their widespread presence in urban environments and frequent contact with wild birds make them potential reservoirs and vectors of resistant pathogens [7]. Pigeons have been identified as potential carriers of more than 60 zoonotic pathogens [8]. Among them, *Staphylococcus* species are of particular concern, as they can infect both humans and animals, posing significant public health risks through potential zoonotic transmission. Although human infections directly linked to pigeon-derived *S. aureus* are rare, a recent case report described a multidrug-resistant *S. aureus* strain isolated from a pigeon environment in close proximity to an immunocompromised patient, raising concerns about indirect zoonotic transmission and environmental contamination [9]. Moreover, air sampling studies conducted at pigeon exhibitions have detected airborne methicillin-resistant *S. aureus* (MRSA) strains, suggesting a potential inhalational exposure risk for humans in close contact with pigeon flocks [10]. Consequently, it is important to reduce antibiotic use in pigeon husbandry [11,12,13,14,15,16,17,18,19], and improve biosecurity and pharmacological practices [20,21].

This study focuses on *S. aureus*, a genus which includes over 30 species with zoonotic relevance [22]. These Gram-positive, non-spore-forming, non-flagellated cocci often exhibit alpha- or beta-hemolysis on blood agar [23]. The most common species in pigeons include *S. aureus*, *S. delphini*, and *S. intermedius* [9]. In poultry, *S. aureus* infections typically cause arthritis [24] and dermatitis [25]. In pigeons kept under poor hygienic conditions, pododermatitis may develop, potentially progressing to osteomyelitis and digital necrosis [23]. These infections are typically treated with beta-lactam antibiotics such as amoxicillin, amoxicillin-clavulanate, or cephalosporins [26]. However, resistance mechanisms, including beta-lactamase production and the emergence of MRSA and methicillin-resistant *S. pseudintermedius* (MRSP), are increasingly common [27,28]. In light of these findings, *S. aureus* isolated from pigeons may represent not only a veterinary concern, but also a potential source of community- or environment-associated human infections [9,10].

Amoxicillin is commonly used to treat *S. aureus* infections in pigeons [26,29]. It is a semi-synthetic penicillin [30] that exerts its bactericidal activity by inhibiting bacterial cell wall synthesis [31]. To improve its efficacy, amoxicillin is routinely used with clavulanic acid in human medicine. However, clavulanic acid, while enhancing amoxicillin’s efficacy against beta-lactamase-producing strains [32,33], is not approved for use in poultry, due to the lack of an established maximum residue limit (MRL) [34,35].

As *Staphylococcus* and *Streptococcus* species are generally considered secondary pathogens in pigeons, tiamulin has been used in treatment [36] with good absorption via feed or water [37]. Tiamulin [38], a pleuromutilin antibiotic, is widely used in veterinary medicine. Like amoxicillin, it also inhibits protein synthesis and is effective [39] against *Mycoplasma* [40,41] and other respiratory pathogens [42].

The aim of the present study was to determine the antimicrobial susceptibility profiles of *S. aureus* isolates collected from pigeons in Hungary in 2022. The results provide insights into the role of pigeons in AMR dissemination and contribute to the development of improved veterinary treatment strategies.

## 2. Results

**Figure 1:**
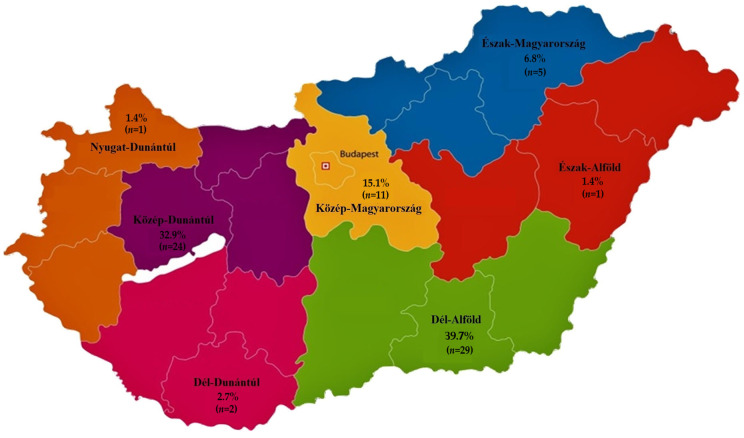
Regional distribution of *Staphylococcus aureus* isolates from pigeons (*n* = 73), including national distribution by usage type, age group, and flock size.

A total of 73 *S. aureus* isolates from pigeons were tested for antimicrobial susceptibility. Most isolates originated from the Dél-Alföld region (39.7%) and the Közép-Dunántúl region (32.9%). The majority of the isolates were from racing pigeons (56.2%), with young adults representing the largest age group (35.6%). Furthermore, 50.7% of the pigeon owners maintained medium-sized flocks (51–100 birds). The regional distribution, usage types, age groups, and flock sizes are illustrated in Figure 1.

**Figure 2:**
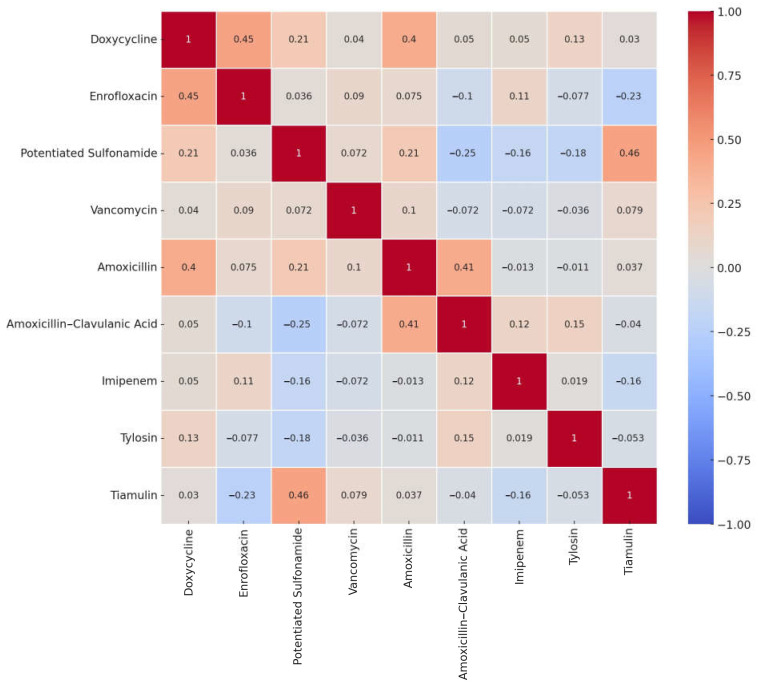
Correlation analysis of *Staphylococcus aureus* isolates from pigeons (*n* = 73) illustrated using a heatmap. A heatmap of pairwise Pearson correlation coefficients (r) between antimicrobial agents, based on their binary resistance profiles across all *E. coli* isolates. The r values range from −1 to +1, where +1 indicates a perfect positive correlation (resistance to both agents tends to co-occur), 0 indicates no linear relationship, and −1 indicates a perfect negative correlation (resistance to one agent is associated with susceptibility to the other). Correlations were calculated using binary resistance data (1 = resistant, 0 = susceptible) based on clinical breakpoints.

The correlation analysis examined the relationships between different antimicrobial resistances (Figure 2). A Pearson correlation matrix was used to quantify the associations between antibiotics, which may have emerged due to shared mechanisms of action or simultaneous use. The heatmap provides a comprehensive overview of potentially significant association patterns. Notably, a strong positive correlation was observed between potentiated sulfonamide and tiamulin (0.46), enrofloxacin and doxycycline (0.45), and amoxicillin and amoxicillin-clavulanate (0.41). These correlations suggest that some antibiotics may be commonly used together, contributing to the development of cross-resistance.

The identification of multidrug-resistant (MDR), extensively drug-resistant (XDR), and pandrug-resistant (PDR) strains revealed that 80.8% (*n* = 59) of the *S. aureus* strains were MDR, 17.8% (*n* = 13) were XDR, and 1.4% (*n* = 1) were PDR. The high prevalence of MDR strains and their association with particular antibiotics, such as doxycycline and tylosin, suggest that these drugs may be contributing factors to the development of resistance in the isolated strains.

**Figure 3:**
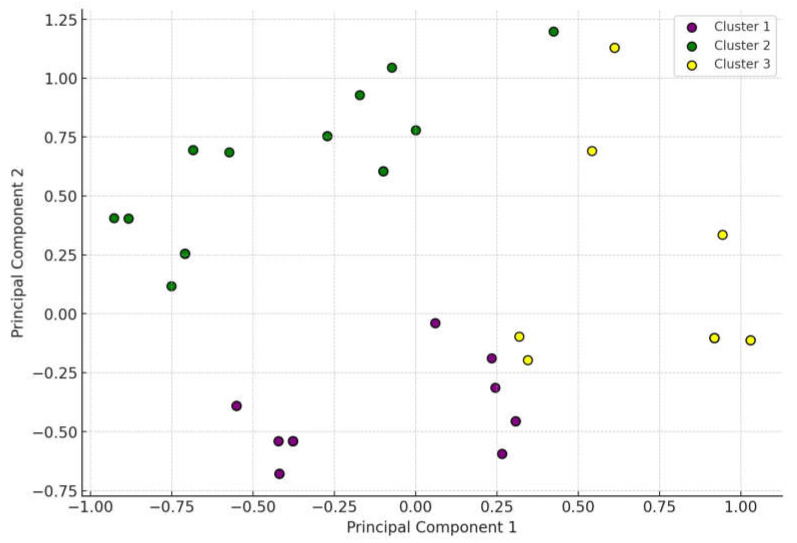
Cluster analysis of *Staphylococcus aureus* isolates from pigeons (*n* = 73). The three main clusters include Cluster 1 (purple) and Cluster 2 (green) dominated by doxycycline-resistant strains, and Cluster 3 (yellow) dominated by tylosin-resistant strains.

The cluster analysis categorized the isolates into three groups exhibiting different dominant antimicrobial resistances (Figure 3). Cluster 1 (purple) and Cluster 2 (green) predominantly displayed resistance to doxycycline, while Cluster 3 (yellow) showed extensive resistance to tylosin. These results indicate that certain groups of *S. aureus* strains exhibit distinct resistance profiles, potentially influenced by the frequent use of particular antibiotics or other factors.

**Figure 4:**
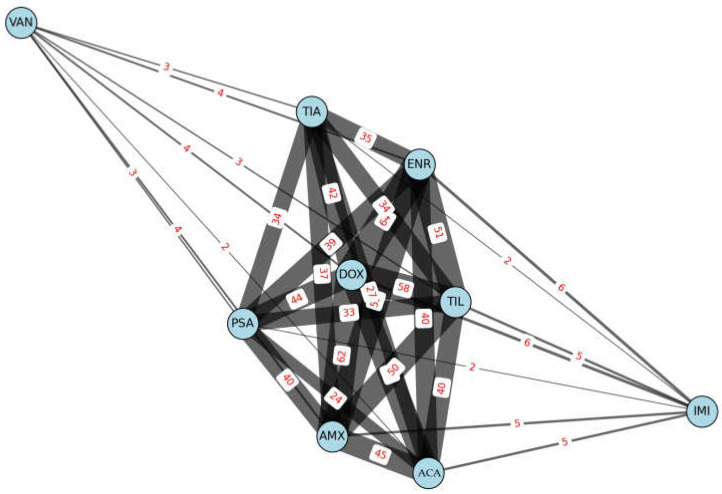
A network graph illustrating the resistance profile of *Staphylococcus aureus* isolates from pigeons (*n* = 73). AMX—amoxicillin; ACA—amoxicillin-clavulanic acid; DOX—doxycycline; ENR—enrofloxacin; IMI—imipenem; PSA—potentiated sulfonamide; TIL—tylosin; VAN—vancomycin; TIA—tiamulin.

The network graph analysis illustrated the relationships between antibiotics based on their co-occurring resistance patterns (Figure 4). Antibiotics are represented by light blue circles, with connection thickness indicating the frequency of co-occurring resistance. The most commonly associated antibiotic was doxycycline, frequently displaying co-resistance with amoxicillin, amoxicillin-clavulanate, and tylosin. The frequent co-occurrence of doxycycline and tylosin resistance may indicate a cross-resistance mechanism, which could have implications for treatment strategies involving these antibiotics.

**Figure 5:**
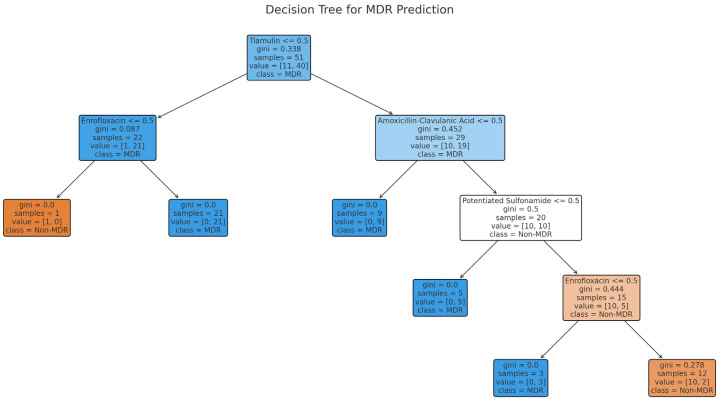
Decision tree predicting multidrug resistance in *Staphylococcus aureus* isolates from pigeons (*n* = 73).

Using a decision tree model, we attempted to predict the presence of MDR strains (Figure 5). The most significant variables influencing resistance were tiamulin, enrofloxacin, and amoxicillin-clavulanate. The model achieved an accuracy of 86.5%, with a precision of 94.4%, sensitivity of 89.5%, and an F1 score of 91.9%. These results demonstrate that specific antibiotics contribute significantly to predicting MDR strains, highlighting the importance of monitoring their usage to prevent resistance.

**Figure 6:**
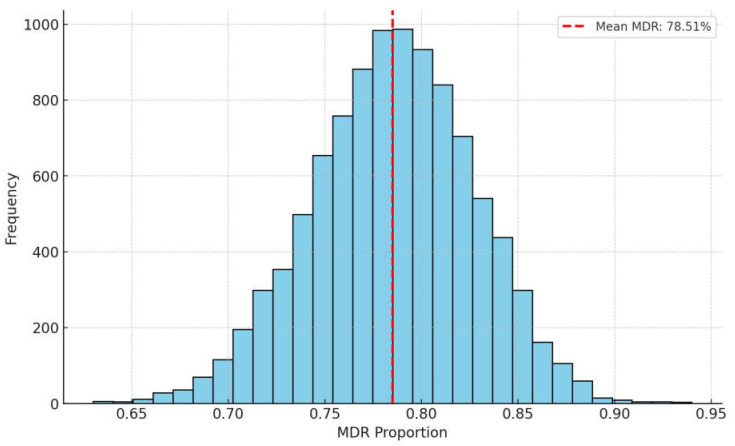
Monte Carlo simulation predicting multidrug-resistant strains in *Staphylococcus aureus* isolates from pigeons (*n* = 73).

A Monte Carlo simulation was conducted to estimate the probability of MDR *S. aureus* strains based on the available data (Figure 6). The simulation consisted of 10,000 iterations, each analyzing the results of 100 samples. The mean MDR occurrence was 78.51%, supported by the median value of the histogram, indicating a symmetrical distribution around the mean. The range of distribution varied from approximately 0.65 to 0.95, suggesting that while MDR strains dominate, non-MDR strains can still be present. The results of the Monte Carlo simulation highlight the need for targeted surveillance and improved antimicrobial stewardship to reduce the prevalence of MDR strains in pigeons.

Following the determination of minimum inhibitory concentration (MIC) values, a frequency table was prepared for each substance (Table 1). MIC_50_ and MIC_90_ values were calculated and compared with clinical breakpoints and epidemiological cutoff values (ECOFF) provided by the European Committee on Antimicrobial Susceptibility Testing (EUCAST). For imipenem, at least 90% of the population remained susceptible (MIC_90_ = 4 µg/mL), which was also true for vancomycin (MIC_90_ = 16 µg/mL). However, considering the ECOFF values, less than 50% of the population were classified as wild-type strains for all examined antibiotics. The observation that imipenem and vancomycin maintain high efficacy against most isolates suggests that these antibiotics could be valuable treatment options for severe infections, although their use should be carefully monitored to prevent resistance development.

Detailed MIC values and isolate-specific data are available in the Supplementary Materials.

**Figure 7:**
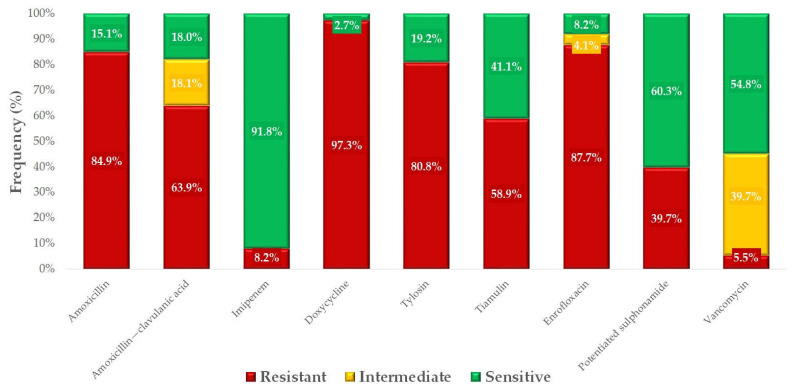
Antimicrobial susceptibility profiles of *Staphylococcus aureus* isolates (*n* = 73) from pigeons for antibiotics of public and animal health significance.

We determined the proportion of susceptible and resistant strains for each active ingredient based on clinical breakpoints (Figure 7). Remarkably high resistance was detected for doxycycline (97.3%), enrofloxacin (87.7%), and amoxicillin (84.9%). The lowest resistance levels were observed against vancomycin (5.5%) and imipenem (8.2%).

**Figure 8:**
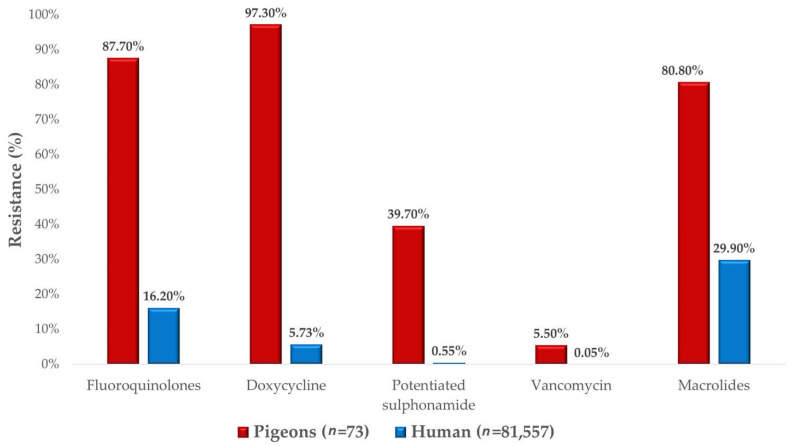
Comparison of resistance values between *Staphylococcus aureus* isolates from pigeons and human resistance data.

Comparison with human resistance data was also possible (Figure 8). The results demonstrate that resistance rates were generally higher in the veterinary sector than in public health settings. An exception was observed with vancomycin, which showed low resistance levels in both human and veterinary samples.

## 3. Discussion

The aim of the present study was to comprehensively investigate the antimicrobial resistance patterns of *S. aureus* strains isolated from pigeons using various statistical and predictive methods. Our analysis involved correlation studies, cluster analysis, network graph analysis, decision tree modeling, and stochastic modeling via Monte Carlo simulation to estimate the prevalence and potential spread of antibiotic-resistant strains.

A total of 74 *S. aureus* strains were examined. Following the determination of the MIC_90_ values, imipenem (4 µg/mL) and vancomycin (16 µg/mL) demonstrated that at least 90% of the investigated population retained sensitivity to these agents. In 2005, an Italian study on samples taken from pigeons reported that all strains were susceptible to amoxicillin-clavulanate, potentiated sulfonamides, and vancomycin, with an enrofloxacin resistance rate of 20.3% [43]. In contrast, our findings revealed resistance rates of 63.9% for amoxicillin-clavulanate, 39.7% for potentiated sulfonamides, 5.5% for vancomycin, and 87.7% for enrofloxacin. In 2014, a comprehensive Polish study reported resistance rates of 12% for amoxicillin, 3% for amoxicillin-clavulanate, 75% for enrofloxacin, 43% for doxycycline, and 2% for potentiated sulfonamides in samples taken from pigeons [44]. Our study, however, found resistance rates of 84.9%, 63.9%, 87.7%, 97.3%, and 39.7% for the same antibiotics, respectively. Additionally, a separate study conducted in 2008 reported resistance rates of 3.7% for enrofloxacin and 32.1% for tylosin in poultry [45], whereas our study found resistance rates of 57.7% and 80.8%, respectively.

In this study, we also investigated the prevalence of MDR *S. aureus* strains. Our results indicate an alarmingly high rate of MDR strains (80.8%), highlighting pigeons’ potential role as reservoirs of MDR bacteria. The results of the Monte Carlo simulation further confirmed that the proportion of MDR strains exceeded 78% in a significant portion of the samples, supporting the dominant presence of these pathogens within the examined population. Similar findings were observed in a study conducted in South Africa, where 87.7% of the isolated *S. aureus* strains from wild pigeons were found to be MDR, with 84.2% resistance to penicillin and 82.5% resistance to ciprofloxacin. This reinforces the potential role of pigeons in the dissemination of antibiotic-resistant pathogens within the environment, because wild pigeons travel long distances and come into contact with a lot of other birds [46].

Additionally, a case report from Poland documented the isolation of multiple MDR pathogens, including *S. aureus* strains displaying methicillin resistance, from the oral cavity of a racing pigeon. This case highlights the potential role of pigeons in directly transmitting MDR bacteria through close contact with humans [9].

Furthermore, an Italian study conducted on *S. aureus* strains from a pigeon slaughterhouse environment revealed that these bacteria could exhibit resistance to multiple antibiotic groups, further confirming the potential role of pigeons in the spread of MDR strains [43].

These findings clearly suggest that pigeons can act as significant reservoirs for MDR pathogens, contributing to the dissemination of resistant bacteria within both natural and urban environments. Additionally, our findings emphasize the need for coordinated, comprehensive research and monitoring programs aimed at accurately mapping the spread of these pathogens in the environment. Moreover, this study opens new avenues for research focused on multi-drug resistance in pigeons, which should encompass not only wild and urban populations but also flocks associated with pigeon breeding and racing activities.

Although the isolates were obtained from multiple geographic regions across Hungary, we did not perform region-based statistical analysis, due to the highly uneven distribution of sample numbers among locations. In certain areas, only a few isolates were available, which could have introduced significant bias or overinterpretation. Nevertheless, evaluating the potential geographic variation in resistance patterns remains an important objective, and future studies with larger and more balanced regional sample sizes are planned to address this question.

These findings underline the need for targeted public health measures, including the routine monitoring of antimicrobial resistance in synanthropic avian populations. Given the close proximity of pigeons to humans in urban settings, policies aiming to regulate antimicrobial use in pigeon husbandry, improve hygienic conditions, and raise awareness among fanciers may help mitigate the zoonotic transmission risk of multidrug-resistant bacteria. One Health-based surveillance strategies should explicitly consider pigeons as potential reservoirs in AMR control programs.

Future studies should include the genetic characterization of resistant strains, investigation of their potential zoonotic transmission, and the development of effective preventive strategies. Given that antimicrobial resistance is becoming an increasingly global health issue, the further exploration of pigeons’ association with MDR strains is essential from both public health and veterinary perspectives. It is particularly crucial to examine whether pigeons can act as vectors in the One Health context, potentially transmitting resistant strains to humans and other animals. Additionally, further research is needed to assess whether the antimicrobial resistance profiles identified in pigeons are linked to specific agricultural practices or urban habitats, and how these findings could guide the development of more effective surveillance and intervention strategies.

## 4. Materials and Methods

### 4.1. Origin of Strains and Human Data

The strains examined were isolated in 2022. A licensed field veterinarian providing regular health care to domestic pigeon flocks in Hungary collected oropharyngeal and cloacal swab samples during routine diagnostic visits, without prior antimicrobial treatment. Sampling was non-invasive and performed in accordance with standard veterinary diagnostic procedures. A total of 22 flocks were sampled across seven Hungarian regions: Észak-Magyarország (3 flocks), Észak-Alföld (2), Dél-Alföld (8), Közép-Magyarország (5), Közép-Dunántúl (1), Dél-Dunántúl (1), and Nyugat-Dunántúl (2). Swabs were obtained using Amies-type aluminum transport swabs without charcoal (Biolab Zrt., Budapest, Hungary). Samples were transported under refrigeration and processed within 24 h. Strain isolation and preliminary identification were performed using ChromoBio Coliform agar (Biolab Zrt., Budapest, Hungary) according to the manufacturer’s protocol. Pure cultures were provided to our laboratory and stored in Microbank™ cryovials (Pro-Lab Diagnostics, Richmond Hill, ON, Canada) at −80 °C for downstream analysis. This approach is consistent with previous methodologies used in avian microbiological surveillance studies [47].

The human resistance data were provided by the Hungarian National Public Health Center. The human resistance data focused on ampicillin, whereas amoxicillin was considered for veterinary cases. For third-generation cephalosporins, comparisons were made against ceftriaxone. Aminoglycoside resistance data included gentamicin, tobramycin, and amikacin, along with additional individual data for neomycin. Similarly, fluoroquinolone resistance was analyzed collectively, while enrofloxacin was specifically assessed for veterinary cases. The human resistance data, including both aggregated and region-specific information, were provided with permission from the National Chief Medical Officer in an Excel file. The dataset contained resistance rates expressed as percentages.

### 4.2. Determination of Minimum Inhibitory Concentration (MIC)

The phenotypic expression of resistance was assessed by determining MIC values according to the guidelines of the Clinical Laboratory Standard Institute (CLSI) [48]. The breakpoints were also defined following CLSI guidelines [48] and compared with the epidemiological cutoff value (ECOFF) established by the European Committee on Antimicrobial Susceptibility Testing (EUCAST) [49]. For antimicrobial agents lacking CLSI-defined breakpoints, such as imipenem [50], tylosin [51], and tiamulin [52], we relied on published data. MICs were determined once per isolate using the standard microdilution methodology, without technical replication. Therefore, MIC values represent single measurements per isolate. Statistical analyses were based on resistance categorization, MIC_50_ and MIC_90_ summary values, and frequency-based metrics rather than mean ± SD calculations.

The bacterial strains stored at −80 °C were suspended in 3 mL of cation-adjusted Mueller–Hinton broth (CAMHB) and incubated at 37 °C for 18–24 h before testing. The assays were performed using 96-well microtiter plates (VWR International, LLC., Debrecen, Hungary). Except for the first column, all wells were filled with 90 µL of CAMHB. The stock solutions of the antimicrobial agents (Merck KGaA, Darmstadt, Germany) were prepared at a concentration of 1024 µg/mL according to CLSI guidelines [53].

Amoxicillin and amoxicillin-clavulanate were prepared in a 2:1 ratio (pH 7.2, 0.01 mol/L) and imipenem in phosphate buffer (pH 6, 0.1 mol/L). Doxycycline, neomycin, tylosin, and vancomycin were dissolved in distilled water. Potentiated sulfonamide (trimethoprim and sulfamethoxazole at a 1:19 ratio) was prepared by dissolving sulfamethoxazole in hot water with a few drops of 2.5 mol/L of NaOH, while trimethoprim was dissolved in distilled water containing 0.05 mol/L of HCl. Enrofloxacin was prepared by dissolving it in distilled water with a few drops of 1 mol/L of NaOH. Florfenicol was prepared using a few drops of 95% ethanol mixed with distilled water.

From the diluted 512 µg/mL solution prepared with broth, 180 µL was pipetted into the first column of the microtiter plates, and a twofold serial dilution series was performed. After reaching the 10th column, the excess 90 µL was discarded, leaving 90 µL in each well. Bacterial suspensions adjusted to 0.5 McFarland standard using a nephelometer (ThermoFisher Scientific, Budapest, Hungary) were inoculated into the microtiter plates from the 11th column backward, adding 10 µL per well [54].

Evaluation was performed using a Sensititre™ SWIN™ automatic MIC reader (ThermoFisher Scientific, Budapest, Hungary) and VIZION system software version 3.4 (ThermoFisher Scientific, Budapest, Hungary, 2024). The quality control strain used was *S. aureus* (ATCC 23235).

### 4.3. Statistical Analysis

Statistical analyses were performed using R programming language version 4.2.2 within the RStudio version 2022.12.0+353 environment [55]. Normality was assessed using the Shapiro–Wilk test, and non-parametric tests were applied for data that did not follow a normal distribution. The Kruskal–Wallis test [56] was applied to compare resistance levels across various factors. This test does not assume a normal distribution and is suitable for comparing medians across multiple sample groups, making it ideal for analyzing differences between categories. Post hoc testing was then performed to determine the precise relationships between groups. For this purpose, the Mann–Whitney U test [57] and t-test were applied, comparing all categories pairwise, followed by Bonferroni correction to adjust for the inflated *p*-values resulting from multiple comparisons [58]. It is important to note that applying the Bonferroni correction may increase the risk of Type II errors (failing to detect real differences).

Heat map analysis was used to examine correlations between different antibiotics, employing the corrplot (v0.92) and pheatmap (v1.0.12) packages.

For cluster analysis, hierarchical clustering was performed, and visualizations were created using the factoextra (v1.0.7) package. Agglomerative hierarchical clustering was carried out using the cluster (v2.1.4) package, while dendrograms were visualized with the dendextend (v1.16.0) package.

Network analysis was conducted to investigate cross-resistance patterns between different antibiotics. Graphs were constructed and analyzed using the igraph (v1.3.5) package, and data visualization was performed with the ggraph (v2.1.0) package.

Decision tree modeling was performed to predict MDR strains, with model construction using the rpart (v4.1.16) package, performance evaluation with the caret (v6.0.93) package, and visualization of the decision tree with the rpart.plot (v3.1.2) package.

The prevalence of MDR strains was estimated using Monte Carlo simulation. Bootstrap sampling with 10,000 iterations was conducted using the boot (v1.3.28) package, and the aggregation of results and calculation of statistical metrics were performed using the dplyr (v1.1.0) package. Visualization of the simulation results was carried out using the ggplot2 (v3.4.0) package.

The analyses were performed using open-source R packages, ensuring full reproducibility.

## 5. Conclusions

The findings of the present study clearly demonstrate that pigeons may serve as a significant reservoir for MDR *S. aureus* strains. The remarkably high MDR prevalence rate of 80.8% strongly supports the potential role of pigeons in the environmental dissemination of antibiotic-resistant bacteria.

The results obtained from the Monte Carlo simulations further suggest that the predominance of MDR strains is not a random occurrence, but rather part of a broader epidemiological pattern that requires increased attention in future research.

Our results highlight the urgent need for genetic characterization of MDR isolates, continuous monitoring of their environmental spread, and the development of effective control strategies. Given that antimicrobial resistance poses a growing global health threat, it is particularly important to understand the extent to which pigeon populations contribute to the persistence and transmission of these pathogens.

This study may serve as a foundation for a broader research program aimed at a more comprehensive understanding of the biological, ecological, and epidemiological roles of pigeon-associated MDR strains.

## Figures and Tables

**Table 1:** A frequency distribution table of minimum inhibitory concentrations (MICs) for *Staphylococcus aureus* isolates (*n* = 73) from pigeons, tested against antibiotics with established clinical breakpoints. The upper row represents the frequency values, while the lower row indicates the corresponding percentage. Vertical red lines denote the clinical breakpoints, while vertical green lines represent the epidemiological cutoff values (ECOFF) defined by the European Committee on Antimicrobial Susceptibility Testing (EUCAST). The grey colour is used to denote the empty cells at the edges, it is quite common, it is used consistently in many publications and nowhere does it need to be explained.

Antibiotics	Breakpoint	0.001	0.002	0.004	0.008	0.016	0.031	0.063	0.125	0.25	0.5	1	2	4	8	16	32	64	128	256	512	1024	MIC_50_	MIC_90_	^3^ ECOFF
	µg/mL																								
Amoxicillin	0.5								6	5	7	8	6	11	9	6	1	14					4	64	0.5
									8.2%	6.8%	9.6%	11.0%	8.2%	15.1%	12.3%	8.2%	1.4%	19.2%							
^1^ Amoxicillin-clavulanic acid	1						1	3	2	7	13	13	14	9	6	4							1	8	0.5
							1.4%	4.1%	2.7%	9.6%	17.8%	17.8%	19.2%	12.3%	8.2%	5.5%									
Doxycycline	0.5							2	0	0	0	6	2	6	10	23	14	6	4				16	64	0.5
								2.7%	0.0%	0.0%	0.0%	8.2%	2.7%	8.2%	13.7%	31.5%	19.2%	8.2%	5.5%						
Enrofloxacin	4							4	1	0	1	2	1	4	10	8	42						32	32	0.5
								5.5%	1.4%	0.0%	1.4%	2.7%	1.4%	5.5%	13.7%	11.0%	57.5%								
Imipenem	8						10	11	13	7	4	2	1	19	2	1	3						0.25	4	0.125
							13.7%	15.1%	17.8%	9.6%	5.5%	2.7%	1.4%	26.0%	2.7%	1.4%	4.1%								
^2^ Potentiated sulphonamide	4									2	1	14	12	5	22	10	2	2	2	1			8	16	0.25
										2.7%	1.4%	19.2%	16.4%	6.8%	30.1%	13.7%	2.7%	2.7%	2.7%	1.4%					
Tiamulin	4							8	7	2	2	7	4	3	2	8	6	5	19				16	128	2
								11.0%	9.6%	2.7%	2.7%	9.6%	5.5%	4.1%	2.7%	11.0%	8.2%	6.8%	26.0%						
Tylosin	64									1	1	1	3	1	4	1	2	2	57				128	128	2
										1.4%	1.4%	1.4%	4.1%	1.4%	5.5%	1.4%	2.7%	2.7%	78.1%						
Vancomycin	32								1	0	5	10	10	14	13	16	4						4	16	2
									1.4%	0.0%	6.8%	13.7%	13.7%	19.2%	17.8%	21.9%	5.5%								

^1^ ratio of 2:1; ^2^ trimethoprim and sulfamethoxazole in a 19:1 ratio; ^3^ epidemiological cutoff values (ECOFF) defined by the European Committee on Antimicrobial Susceptibility Testing (EUCAST).

## Data Availability

The data presented in this study are available from the corresponding author upon reasonable request.

## Supplementary Materials

The following supporting information can be downloaded at https://www.mdpi.com/article/10.3390/antibiotics14050525/s1. Additional data excel file.

## Abbreviations

The following abbreviations are used in this manuscript:

AMR	Antimicrobial resistance
CAMHB	Cation-adjusted Mueller Hinton Broth
CLSI	Clinical Laboratory Standards Institute
ECOFF	Epidemiological cutoff values
EUCAST	European Committee on Antimicrobial Susceptibility Testing
MDR	Multidrug-resistant
MIC	Minimum inhibitory concentration
PDR	Pandrug-resistant
WHO	World Health Organization
XDR	Extensively drug-resistant
